# Discovery of a Novel DYRK1A Mutation (c.524del) in Intellectual Development Disorder Autosomal Dominant 7 (MRD7): A Comprehensive Case Analysis

**DOI:** 10.1155/2024/2926555

**Published:** 2024-07-30

**Authors:** Fiona Whitaker, Alvaro Serrano

**Affiliations:** East Tennessee State University, Johnson City, TN, USA

## Abstract

Dual-specificity tyrosine kinase 1A (DYRK1A) is a member of the CMGC family that is linked to a multitude of neuronal development pathways. Both overexpression and insufficiency of this gene are associated with many recognizable disorders, including Down syndrome and DYRK1A-related intellectual disability syndrome which is characterized by distinct physical features with microcephaly and global developmental delay. We report a case of DYRK1A-related intellectual disability syndrome caused by a novel mutation.

## 1. Introduction

DYRK1A mutations have been linked to multiple neurological disorders including Down syndrome, intellectual development disorder autosomal dominant 7 (MRD7), dementia, Alzheimer's disease, fronto-temporal degeneration, Huntington's disease, and Parkinson's disease. This is due to the gene's extensive involvement in neuronal development and signaling pathways. DYRK1A is located in human chromosome 21q22.2, known as the Down syndrome critical region (DSCR). Activation of DYRKs depends on the autophosphorylation of a tyrosine residue in the activation loop [1]. Mutations lead to DYRK1A proteins that lack all or part of the kinase domain and are catalytically inactive [2]. Overexpression of DYRK1A is strongly associated with Down syndrome, whereas haploinsufficiency is associated with MRD7. Mutations that result in haploinsufficiency include microdeletions, translocations, single nucleotide variants, or insertions/deletions [3, 4].

The phenotypic presentation of these patients usually includes microcephaly, intellectual disability, speech delay, facial dysmorphisms, seizures, vision abnormalities, and feeding difficulties. The facial dysmorphisms stay consistent across affected individuals, with MRD7 patients having posteriorly rotated and protruding ears with thick and overfolded helices, prominent eyebrows, high anterior hairlines, and retrognathia. Patients can also have foot abnormalities with proximal placement of the first toe, crooked toes, and syndactyly of the second and third toes [5].

Here, we present the case of a patient with a clearly pathogenic mutation in DYRK1A that has not been described in the literature.

## 2. Clinical Case Presentation

**Figure 1:**
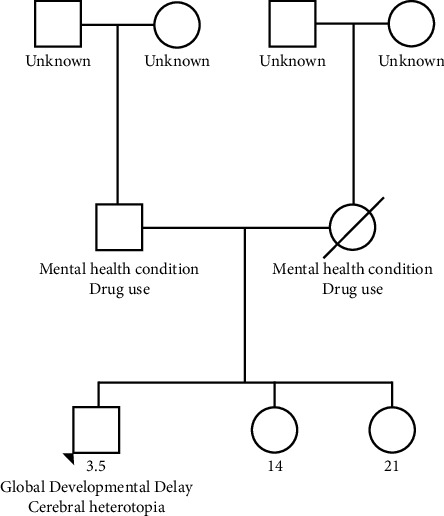
Pedigree limited as the patient is adopted.

A 3.5-year-old male was referred for the first time to a medical genetics clinic by his pediatrician for developmental delay associated with cortical nodular heterotopia on brain imaging. Maternal drug screening was positive for buprenorphine. He was born via spontaneous vaginal delivery at 40 weeks with APGARs of 7 and 9 at 1 and 5 minutes, respectively. At birth, his neonatal abstinence score was 17 and he was placed on morphine replacement therapy. He also had poor feeding while in the neonatal intensive care unit. Auditory brainstem response testing was normal at birth. Metabolic newborn screen was normal. Circumcision was performed on day of life 5. Birth weight was 3425 g, and discharge weight was 3195 g. At 2 months of age, his height was below 1 percentile and normal motor development with borderline delayed speech development. At this visit, he was noted to have retrognathia, externally rotated ears, and a smooth philtrum. He had consistently poor follow-up due to social factors. Patient was in foster care and then adopted, so family history is limited (Figure 1).

At his 8-month checkup, he was noted to have delayed communication, gross motor, fine motor, problem-solving, and personal-social skills. He was found to have consistent deep airway penetration and airway aspiration with a negative cough response on barium swallow and a concern for hearing impairment. Referrals were placed to audiology, speech therapy, physical therapy, and occupational therapy at this time. Chest x-ray obtained for aspiration concerns revealed possible right atrial and ventricular enlargement and cardiology consulted. Echocardiogram showed no congenital abnormalities.

He presented to the emergency department at 15 months old with a febrile seizure that resolved, and he was discharged the same day. At 22 months, he was evaluated and showed a medium risk of autism based on the modified checklist for autism in toddlers. He was admitted to the hospital at 2 years of age with multiple generalized tonic-clonic seizures, and a head CT showed prominent lateral and third ventricles. Neurology was consulted, and he was started on levetiracetam. During his follow-up with neurology, his mom reported a history of staring spells daily lasting up to a few minutes where he would not interact with her if she tried to get his attention. Levetiracetam was increased and trileptal started. A brain MRI at age 2.5 years showed nodular gray matter heterotopia in the posterior right temporal lobe adjacent to the trigone of the right lateral ventricle and in the left posterior frontal lobe gray-white matter junction. The cerebral ventricles were also mildly prominent in size. A swallow study at 3 years old showed flash airway penetration with thin liquids and had subsequent G tube placement. He has received all age-appropriate vaccinations.

**Figure 2:**
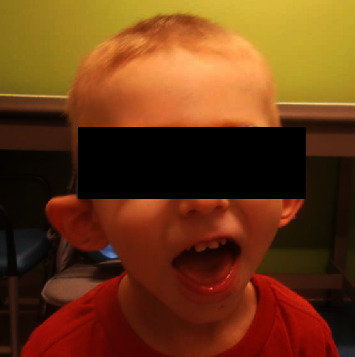
Characteristic facial features. Microcephaly, broad and tall forehead, posteriorly rotated low-set ears, thin upper lip, and pointed chin.

**Figure 3:**
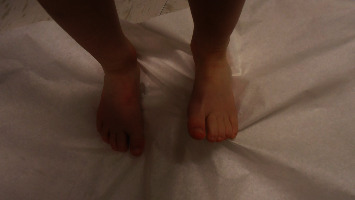
Foot abnormalities present in this patient.

**Figure 4:**
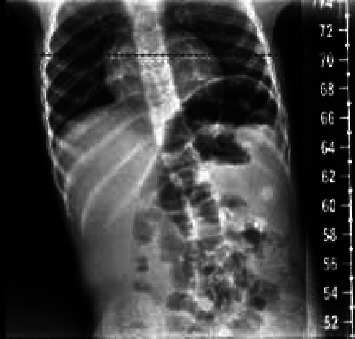
Scoliosis found on X-ray.

He was seen by genetics for the first time at 3.5 years. At this initial visit, his weight was 1 percentile, height was 10 percentile, body mass index (BMI) was 1 percentile, and head circumference was <3 percentile with global developmental delay. The patient is unable to speak at this point. He ambulates independently but has frequent falls. On exam, he had microcephaly with a broad and tall forehead, low set, posteriorly rotated ears with a thin upper lip, and pointed chin (Figure 2). The patient presented with foot abnormalities (Figure 3). Renal ultrasound normal and spine x-ray showed approximately 15 degree levoscoliosis of the lumbar spine with its apex at L3 (Figure 4).

## 3. Methods

A peripheral blood sample was collected and sent to GeneDx for analysis. Clinical information detailing characteristics such as developmental delay, cortical nodular heterotopia, autism spectrum disorder, microcephaly, and seizures were included to direct testing. GeneDx performed genomic DNA extraction directly from the specimen, focusing on the complete coding regions and splice site junctions across most of the human genome. The analysis utilized NCBI RefSeq transcripts, and the human genome built GRCh37/UCSC hg19. Sequence variants were reported following guidelines set by the Human Genome Variation Society (HGVS), while copy number variants were reported based on probe coordinates, exon coordinates, or specific breakpoints when available. It was not stated if Sanger sequencing was utilized. No software such as Face2Gene was utilized.

The peripheral blood sample was also sent for whole genome CMA, Fragile X, and mitochondrial genome through GeneDx. At this point, differential diagnosis was broad including metabolic disorders and chromosomal disorders such as 22q11 microdeletion and disorders such as Noonan syndrome, Kabuki syndrome, Smith–Lemli–Opitz and microcephalic dwarfism disorders. CMA, Fragile X, and mitochondrial genome were nondiagnostic. Proband-only exome sequencing (XomeDxPlus) showed a heterozygous pathogenic variant in DYRK1A (NM_001396.3) c.524del; p.Lys175Argfs^*∗*^15 exon 5. The GeneDx report stated “frameshift variant predicted to result in protein truncation or nonsense mediated decay in a gene for which loss of function is a known mechanism of disease. Not observed at significant frequency in large population cohorts (gnomAD). Clinical features described are compatible with the molecular result.”

## 4. Discussion

We presented a case of a 3.5-year-old male with MRD7 caused by a previously unknown mutation in DYRK1A.

Mutation is determined by using reference genome GRCh37/Hg19. gnomAD pLI score 1.00, indicating low tolerance to any loss-of-function mutation, LOEUF 0.27, sHet 0.312, pHaplo 1.00, pTriplo 0.98. DECIPHER HI score 3.69. Pathogenicity frequency in DYRK1A mutations is reported via DECIPHER as pathogenic: 66%, likely pathogenic: 29%, likely benign: 2%, uncertain: 2%, not classified: 2%. Frameshift mutations resulting from deletions in a section of DYRK1A have been reported 55 times in the literature. A single nucleotide variant has been described at this locus previously [6]. 79% of DYRK1A mutations are de novo (with confirmed parentage). These scores support that the majority of mutations in DYRK1A are dosage-sensitive haploinsufficiencies that can significantly affect patients [7–9].

DYRK1A is a highly conserved tyrosine kinase that plays a role in signaling, mRNA splicing, chromatin transcription, DNA damage repair, cell survival, cell cycle control, neuronal development and functions, and synaptic plasticity. Activation of DYRK1A relies on intrinsic phosphorylation of a conserved tyrosine residue. Dephosphorylation does not inactivate DYRK1A once it has been activated, demonstrating its necessity for activation but not for maintenance. Haploinsufficiency results in dysfunction that widely affects the patient, given that DYRK1A normal function is required in a multitude of processes.

Intellectual developmental disorder, autosomal dominant 7 (MRD7) or DYRK1A-related intellectual disability syndrome (OMIM 614014), results in an autism spectrum-like disorder with distinct physical characteristics. These include sparse scalp hair, bitemporal narrowing, deeply set eyes, periorbital fullness, short nose with high nasal root and pointed nasal tip, prominent ears with underdeveloped ear lobes, variations of the philtrum, thin vermillion border of the upper lip, short chin with horizontal crease and/or chin dimple, microcephaly, and foot abnormalities. Patients frequently require a multitude of treatments and therapies including speech, occupational therapy, physical therapy, and management of frequently occurring medical conditions including epilepsy [2]. This is due to the role DYRK1A plays in many processes.

## 5. Conclusion

The identification of a new mutation causing MRD7 adds evidence that haploinsufficiency of DYRK1A results in a recognizable syndrome [10–12].

## Data Availability

No data were used to support the findings of this study.
